# 

**Published:** 1906-08-25

**Authors:** 


CONTENTS.

The Townshend Case - - - - - - - - - - 357
Infantile Mortality - - - - - - - - - - 358
Annotations - - - - - - - - - - - - 359
Medical Opinion and Movement - - - - - 360
Hospital Clinics—
Chronic Suppuration in the Nasal Accessory Sinuses and its Treatment - - - - - - - - - - - - - - - 361
Meeting of the British Medical Association, Toronto - - - - - - - - - - - - 363
Special Analytical and Health Commission on Tinned and Preserved Food - - - - - 366
New Appliances and Things Medical - - - - - 367
The Book World of Medicine and Science - - 368
Ancient Methods of Treatment - - - - - - 369
Hospital Administration—
The Relation of Insanity to Civilisation—I. - - - - 370
New Out-Patients' Department at the Middlesbrough Infirmary - - - - - - - - - - - - - - 371
Morocco and the Canary Islands as Health or Holiday Resorts 372
Social and Poor Law Problems - - - - - - - 373
The Story of the Insane from Year to Year - - - - 374

*NURSING SECTION.*

Notes on News from the Nursing World—Conditions of Nursing in Germany—Hospital Nurses and Vivisection—*Esprit de Corps*—Proposed Cubicles at Preston—A Visiting Nurse Appointed by a Town Council—An Interesting Appointment—The Nightingale Fund—Brave Belfast Nurses—The Late Matron of the Buchanan Hospital, St. Leonards—Gallant Rescue by a Southport Nurse—Queen Alexandra's Imperial Military Nursing Service—The London Association of Nurses, Limited—New Home at West Bromwich—Short Items - - - - - - - - - - - 295-297
The Nursing Outlook - - - - - - - - - 298
Abdominal Surgery - - - - - - - - - - 299
The Nurses' Clinic - - - - - - - - - - 300
Incidents in a Nurse's Life - - - - - - - - 301
The Red Cross Societies' Exhibits at Milan - - 301
Nursing on the Borderland of Afghanistan - - 303
Why Nurses are Scarce in Germany - - - - 304
Everybody's Opinion - - - - - - - - - 305
Appointments - - - - - - - - - - - 305
Presentations - - - - - - - - - - - 305
Practical Hints - - - - - - - - - - - 306
Travel Notes and Queries - - - - - - - 306
Novelties for Nurses - - - - - - - - - 306
A Book and Its Story - - - - - - - - - 307
Notes and Queries - - - - - - - - - - 308
For Reading to the Sick - - - - - - - - 308

